# Gangliosides as Signaling Regulators in Cancer

**DOI:** 10.3390/ijms22105076

**Published:** 2021-05-11

**Authors:** Norihiko Sasaki, Masashi Toyoda, Toshiyuki Ishiwata

**Affiliations:** 1 Research Team for Geriatric Medicine (Vascular Medicine), Tokyo Metropolitan Institute of Gerontology, Tokyo 173-0015, Japan; mtoyoda@tmig.or.jp; 2 Division of Aging and Carcinogenesis, Research Team for Geriatric Pathology, Tokyo Metropolitan Institute of Gerontology, Tokyo 173-0015, Japan

**Keywords:** cancer, ganglioside, signaling, receptor

## Abstract

At the plasma membrane, gangliosides, a group of glycosphingolipids, are expressed along with glycosphingolipids, phospholipids, and cholesterol in so-called lipid rafts that interact with signaling receptors and related molecules. Most cancers present abnormalities in the intracellular signal transduction system involved in tumor growth, invasion, and metastasis. To date, the roles of gangliosides as regulators of signal transduction have been reported in several cancer types. Gangliosides can be expressed by the exogenous ganglioside addition, with their endogenous expression regulated at the enzymatic level by targeting specific glycosyltransferases. Accordingly, the relationship between changes in the composition of cell surface gangliosides and signal transduction has been investigated by controlling ganglioside expression. In cancer cells, several types of signaling molecules are positively or negatively regulated by ganglioside expression levels, promoting malignant properties. Moreover, antibodies against gangliosides have been shown to possess cytotoxic effects on ganglioside-expressing cancer cells. In the present review, we highlight the involvement of gangliosides in the regulation of cancer cell signaling, and we explore possible therapies targeting ganglioside-expressing cancer.

## 1. Introduction

**Figure 1:**
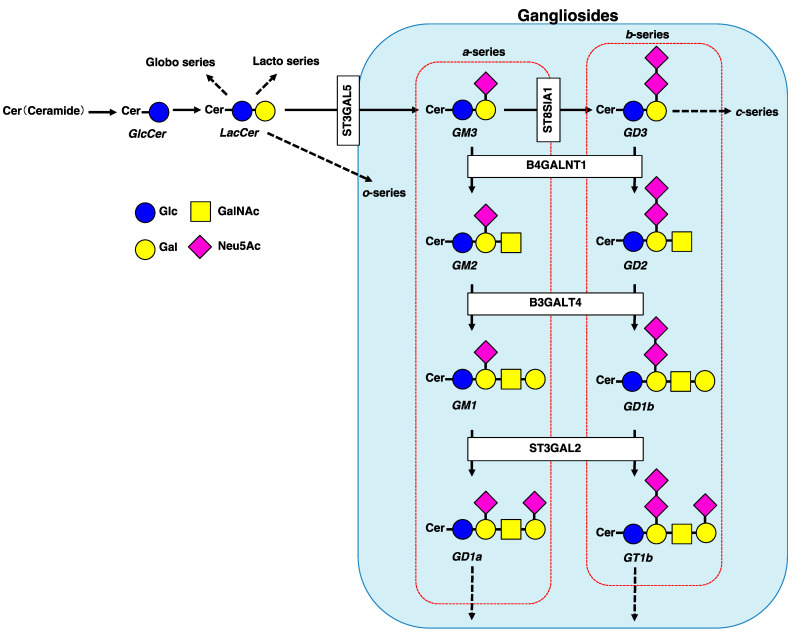
Schematic diagram of major GSL pathways, including gangliosides synthesis glycosyltransferases (ST3GAL5, ST8SIA1, B4GALNT1, B3GALT4, and ST3GAL2). Pathways of the major gangliosides (*a*-series and *b*-series) mentioned in this review are shown within the red dotted rectangles. GSL, glycosphingolipids; Glc, glucose; Gal, galactose; GalNAc, *N*-acetylgalactosamine; Neu5Ac, *N*-acetylneuraminic acid; GlcCer, glucosylceramide; LacCer, lactosylceramide.

Gangliosides are a group of acidic glycosphingolipids (GSLs) possessing one or more sialic acid residues on their carbohydrate moieties, mainly located in sphingolipid and cholesterol-enriched domains called lipid rafts. Raft domains are also enriched with phospholipids and raft-associated proteins [1]. Lipid rafts play important roles in regulating cellular signaling, as well as in physiopathological conditions. Under physiopathological conditions, changes in ganglioside expression levels can affect the localization of raft-associated proteins, resulting in reduced membrane fluidity, followed by cellular dysfunction [2,3,4]. According to the Svennerholm classification, gangliosides can be classified into four series (*o*-, *a*-, *b*-, and *c*-series) based on the number of sialic acid residues (from 0 to 3) linked to the inner galactose residue (Figure 1) [5]. Both *a*-series (GM3, GM2, GM1, and GD1a) and *b*-series (GD3, GD2, GD1b, and GT1b) gangliosides have been well characterized in several types of tissues and cells, including cancer cells [6]. In cancer, the expression levels of these gangliosides are mainly regulated by the availability of glycosyltransferases (Figure 1) and NEU3, a plasma membrane-associated sialidase specific for gangliosides. Typically, cancer cell signaling and development are associated with glycosylation changes, including changes in gangliosides expressed on the cancer cell surface [2,7].

The role of gangliosides as signal regulators was first established by the exogenous addition of gangliosides to a culture medium of HeLa-derivative KB and human epidermoid carcinoma A431 cells [8]. After identification and molecular cloning of ganglioside biosynthetic enzymes, ectopic expression or antisense inhibition targeting specific glycosyltransferases was performed to analyze the role of gangliosides in regulating signal transduction. Receptor tyrosine kinases (RTKs) are activated after ligand binding, inducing receptor dimerization and autophosphorylation of the kinase domain; this results in the activation of diverse signaling cascades that regulate cell survival, proliferation, differentiation, migration, and invasion in various types of cancer. It has been demonstrated that gangliosides are subtle regulators of RTK signaling [9], and that changes in the composition of cancer cell surface gangliosides affect cellular responses [2,6]. Notably, changes in ganglioside expression at the plasma membrane modify the molecular composition and structure of lipid rafts, resulting in the reorganization and/or exclusion of RTKs from lipid rafts [3,4]. The cell surface localization of RTKs correlates with RTK signaling. Reportedly, several RTKs, including receptors for epidermal growth factor (EGF), hepatocyte growth factor (HGF), platelet-derived growth factor (PDGF), or nerve growth factor, are positively or negatively regulated by the expression of gangliosides in cancer cells [2]. Furthermore, gangliosides are involved in apoptosis signaling pathways, such as an extrinsic pathway initiated by CD95 death receptors and an intrinsic pathway through the mitochondria [10]. In lymphoid and myeloid tumor cells, it was first demonstrated that gangliosides could be implicated in apoptosis through the activation of CD95 death receptors and accumulation in mitochondrial membranes, disrupting mitochondrial transmembrane potential and inducing apoptosis in a caspase-independent manner [11]. Notably, the association of gangliosides with cytoskeletal elements, including ezrin, is required to trigger CD95-mediated apoptosis [12]. The localization of the CD95 death receptor in lipid rafts is critical for efficient apoptotic signaling. Accordingly, the structure and composition of ganglioside-containing lipid rafts play a crucial role in the mediation of cell death or survival [10].

In the present review, we highlight the role of gangliosides in cancer cell signaling, including cell growth and apoptosis, and discuss their potential as therapeutic targets for cancer.

## 2. Involvement of Gangliosides in Cancer Cell Signaling

**Figure 2:**
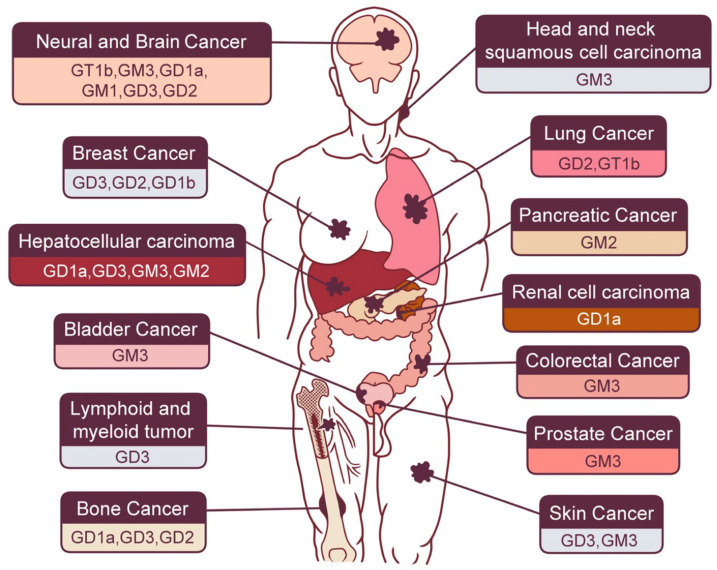
Each species of cancer in the human body and related gangliosides as described in this text.

**Figure 3:**
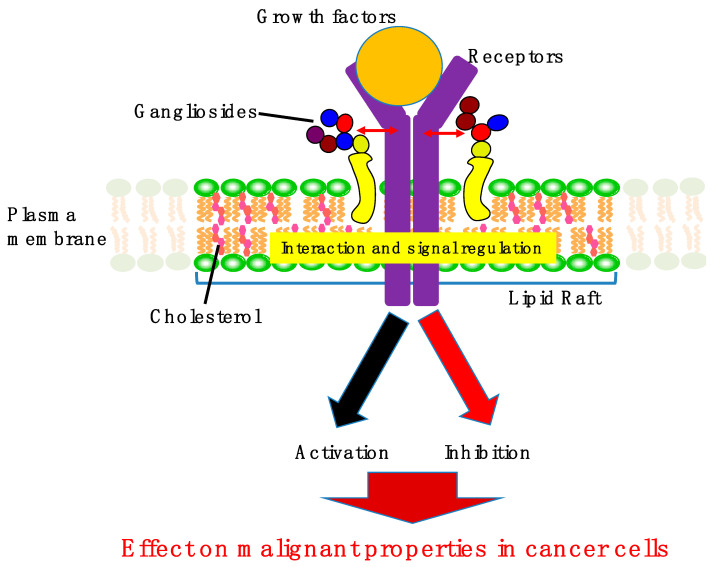
The effects of gangliosides on cancer cell signaling. Gangliosides usually form complexes with several types of RTK receptors in lipid rafts of cancer cells. These interactions contribute to the activation or inhibition of RTK signaling, leading to suppression or promotion of malignant properties in cancer cells. RTK, receptor tyrosine kinase.

As described above, gangliosides are expressed in several types of cancer cells, and their relevance to cancer cell signaling (see Table 1, and Figure 2 and Figure 3) is detailed below.

### 2.1. Gastrointestinal Cancers

#### 2.1.1. Hepatocellular Carcinoma (HCC)

HCC is the sixth most common cancer and the fourth leading cause of cancer-related deaths worldwide [45], and its prevalence is expected to further increase over the next few years. HGF is mainly produced and secreted by stromal cells and contributes to liver regeneration via its high-affinity HGF receptor, c-Met, localized in hepatocytes. HGF binds to c-Met, located on the surface of hepatocytes in a paracrine or autocrine manner. Moreover, the autocrine and paracrine activation of c-Met plays a vital role in HCC development and metastasis [46]. The HGF/c-Met axis promotes the onset, proliferation, invasion, and metastasis of HCC [47]. In human hepatoma HepG2 cells, HGF-induced phosphorylation of c-Met was suppressed after treatment with GD1a. Furthermore, HGF-induced cell scattering in HepG2 cells was inhibited following GD1a treatment [13]. In HepG2 cells, pretreatment with GD3 blocked the nuclear translocation of active κB members, without affecting Akt phosphorylation induced by either treatment. Suppression of κB-dependent gene induction by GD3 was accompanied by enhanced apoptotic cell death induced by these therapies [14]. It has been reported that tetraspanin CD82, a metastasis suppressor, could modulate RTK signaling mediated by gangliosides [48]. In CD82-upregulated mouse HCC Hepa1-6 cells, GM3 alone or GM2/GM3 co-expression enhanced the CD82-mediated inhibition of HGF-induced tyrosine phosphorylation of c-Met at Tyr1313 and Tyr1365 [15]. In contrast, in mouse hepatic cancer cell lines (Hca/A2, Hca/16A3, and Hepa1-6), exogenous GM3 addition enhanced the HGF-induced phosphorylation of c-Met, as well as the activity of the PI3K/Akt signaling pathway. Furthermore, in vitro cell motility and migration were enhanced following HGF stimulation via increased GM3 expression [16]. Reportedly, GM3 inhibits EGF-stimulated motility while promoting HGF-stimulated motility of Hepa1-6 cells via PI3-kinase/Akt-mediated migration signaling [17]. In the highly metastatic murine ascites hepatoma cell line, i.e., HcaF cells, exogenous GM3 addition into the culture medium suppressed phosphorylation of PKB/Akt and EGFR (EGF receptor), as well as inhibited mobility and migration [18].

#### 2.1.2. Pancreatic Cancer

Pancreatic cancer is one of the most lethal malignant tumors, with a 5-year survival rate of approximately 10%. Importantly, pancreatic cancer has been projected as the second leading cause of cancer-related deaths in 2030 [49]. Transforming growth factor (TGF)-β acts as a tumor suppressor in early pancreatic cancer by promoting apoptosis and inhibiting epithelial cell cycle progression while acting as a tumor promoter in the late stage by mediating genomic instability, neoangiogenesis, immune evasion, cell motility, and metastasis [50]. Recent studies have shown that pancreatic cancer is a morphologically and functionally heterogeneous tumor, possessing epithelial-to-mesenchymal features, and TGF-β contributes to the epithelial–mesenchymal transition (EMT) features of pancreatic cancer cells [51,52]. We reported that a glucosylceramide synthase inhibitor, N-(5-adamantane-1-yl-methoxy)-pentyl-1-deoxynojirimycin (AMP-dNM), reduced TGF-β1 signaling, presumably by inhibiting the interaction between GM2 and TGFβ receptor II by downregulating GM2, resulting in suppression of invasion in cancer stem cell (CSC)-like GM2 expressing pancreatic cancer cells [19].

#### 2.1.3. Colorectal Cancer

Colorectal cancer (CRC) is the third most common cancer and the second leading cause of cancer-related deaths worldwide. In addition to the aging population, obesity, low physical activity, and smoking habits are known to increase the risk of CRC. Notably, CRC is a heterogeneous disease, with most cases gradually developing from adenomatous polyps or adenomas [53]. Reportedly, exogenous GM3 treatment dramatically increases cyclin-dependent kinase inhibitor (CKI) p21WAF1 expression by accumulating p53 protein via the PTEN-mediated inhibition of PI-3K/AKT/MDM2 survival signaling, thus resulting in growth inhibition of HCT116 colon cancer cells. Therefore, GM3 represents a modulator of cancer cell proliferation and may have potential use in CRC therapy [20]. Furthermore, exogenous GM3 overexpression sensitized HCT116 cells to cisplatin-induced apoptosis through the cellular accumulation of reactive oxygen species, indicating the involvement of GM3 in the oxidative stress-mediated mitochondrial pathway [21]. NEU3 overexpression enhanced Wnt/β-catenin signaling by accelerating complex formation with LRP6 and recruitment of GSK3β and Axin, facilitating the tumorigenic potential by maintaining the stem-like characteristics of human colon cancer cells; in contrast, its silencing exerted the opposite effects. These effects are considered requisite for modulation of GM3 into lactosylceramide by NEU3 [22].

### 2.2. Neural and Brain Cancer

Neuroblastoma (NB) is a type of cancer formed in nerve tissues and is the third most common cancer in children after leukemia and brain cancer. EGFR expression has been observed in NB tumor specimens [54], as well as in a number of NB cell lines [55]. DaMotta et al. revealed that treatment with the EGFR ligand, EGF, resulted in the proliferation of SK-N-SH NB cells [56]. In the human NB cell line, NBL-W, exogenous addition of GT1b, GM3, or GD1a had an inhibitory effect on EGF-induced EGFR phosphorylation and cell proliferation [23]. Recently, it has been demonstrated that GM1 promotes NB cell differentiation by activating the TrkA receptor via the formation of a TrkA–GM1 oligosaccharide complex at the cell surface [24]. Glioma is a type of tumor that originates in glial cells of the brain or spine [57]. Gliomas are named according to the specific cell type with which they share histological features, and glioblastoma multiforme is a malignant astrocytoma and the most common primary brain tumor in adults. PDGF receptor-α (PDGFRα) is an RTK that is commonly overexpressed and amplified in gliomas [58]. In human glioma cells, GM1 expression by transfection with GM2/GD2 synthase and GM1/GD1b synthase reduced PDGFR phosphorylation and signaling due to the exclusion of the receptor from lipid rafts [25]. GD3 formed a complex with PDGFRα and activated kinase Yes in lipid rafts, thus promoting proliferation, invasion, and a malignant phenotype of human glioma cells [26]. Extracellular signal-regulated kinase (ERK)1/2, a downstream target of the RAS/MAPK (mitogen-activated protein kinase) pathway, is reportedly upregulated in glioma and is involved in cell cycle progression, proliferation, and migration [59]. In the glioma cell line, U-251MG-transfected with GD3 synthase, in which GD3 and GD2 are highly expressed, signaling molecules such as ERK1/2 and Akt are activated, resulting in increased invasion activity and motility [27]. Immunohistochemical analysis revealed that high c-Met expression was associated with poor prognosis in human cases of glioblastoma [60]. In a glioblastoma multiform cell line, GD3 has been suggested to contribute to the self-renewal of CSCs via activation of c-Met signaling [28].

### 2.3. Skin Cancer

The prognosis of patients with advanced melanoma remains poor, with a median overall survival of approximately 8 months and a 5-year overall survival rate of roughly 10% from diagnosis of metastatic disease [61]. The most important and potentially modifiable environmental risk factor for developing malignant melanoma is exposure to ultraviolet (UV) rays, owing to their genotoxic effects [62]. Some of the most important signaling pathways involved in the pathogenesis of melanoma include the MAPK, PI3K/PTEN/AKT, and microphthalmia-associated transcription factor (MITF) signaling pathways [63]. The majority of Yes is localized in lipid rafts in GD3-expressing human melanoma cells. Therefore, upon interaction with GD3, Yes is activated, leading to the malignant phenotype of melanoma cells [29]. In GD3-expressing human melanoma cells, simultaneous treatment with HGF and adhesion to collagen type I resulted in distinct and markedly increased activation of Akt and ERK1⁄2 phosphorylation [30]. Molecules such as p130Cas and paxillin are involved in the GD3-mediated signaling pathways of melanoma cells. Therefore, RNAi knockdown of p130Cas and/or paxillin strongly suppressed GD3-expressing melanoma growth [31]. In other types of skin cancer, endogenous induction of GM3, via transient expression of GM3 synthase gene, reduced EGFR phosphorylation and inhibited cell proliferation in A431 cells (epidermoid carcinoma cells) [32].

### 2.4. Sex Hormone-Related Cancer

Breast and prostate cancers are two common sex hormone-related cancers with high rates of morbidity. Breast cancer is the most common cancer type and the second leading cause of cancer death in women worldwide, expected to account for 29% of all new cancer diagnoses in women [64]. In triple-negative breast cancer [65], accounting for approximately 10–15% of diagnosed breast cancers, expression of estrogen and progesterone receptors is lacking, and the tumor is found to be negative for HER2 overexpression [65,66]. Triple-negative breast cancer constitutes a heterogeneous group of malignancies that are often aggressive and associated with poor prognosis [67]. c-Met overexpression is associated with poor survival rates and cancer activities such as proliferation, migration, and invasion [68]. In MDA-MB-231 breast cancer cells expressing GD3 synthase, the predominantly expressed GD2 is colocalized with c-Met and contributes to the activation of c-Met signaling, resulting in a proliferative phenotype [33]. Furthermore, Liang et al. revealed that GD3 was colocalized and associated with EGFR, activating EGFR signaling, which maintains CSC properties in breast cancer cell lines and breast CSCs [34]. Depleting GD2 by CRISPR knockout of ST8SIA1 attenuated the FAK–AKT–mTOR (mechanistic target of rapamycin) signaling pathway, thus inhibiting the malignant phenotype in triple-negative breast cancer cells [35]. In human breast cancer MCF-7 cells, exogenous GD1b addition or endogenous GD1b production reportedly induced apoptosis through the activation of apoptotic molecules such as caspase-8, caspase-7, and poly (ADP-ribose) polymerase (PARP), without altering the expression of mitochondria-mediated apoptotic molecules [36]. In prostate cancer, NEU3 upregulation activated androgen receptor (AR) signaling by increasing early growth response gene-1, AR, and prostate-specific antigen expression, possibly via EGFR family activation, presumably via modulation of GM3 into LacCer; this can result in androgen-independent cancer cell proliferation [37].

### 2.5. Bone Cancer

Osteosarcomas are the most frequent primary bone sarcomas, predominantly affecting children, adolescents, and young adults [69]. Current therapeutic management, a combined regimen of polychemotherapy and surgery, remains largely insufficient, as patient survival has not improved in recent decades. HGF activates both the mitogen and motogen machinery in osteosarcoma cells [70]. In mouse osteosarcoma cell variant FBJ-LL cells, GD1a inhibited HGF-induced motility and scattering by suppressing c-Met phosphorylation [13]. In osteosarcoma, FAK overexpression and phosphorylation can indicate more aggressive biological behavior and might be an independent predictor of poor prognosis [71]. Moreover, GD3/GD2-positive human osteosarcoma cells showed stronger tyrosine phosphorylation of p130Cas, FAK, and paxillin after serum stimulation than GD3/GD2-negative cells, resulting in malignant properties [38].

### 2.6. Lung Cancer

Small cell lung cancer (SCLC) is the most aggressive form of lung cancer, reportedly accounting for approximately 14% of all lung cancers [72]. Unlike non-small cell lung cancer, in which significant advances have been made using targeted therapies, approved targeted drugs for SCLC are still lacking. Yoshida et al. reported that GD2-expressing SCLC cells exhibited high levels of growth and invasiveness, and the addition of anti-GD2 monoclonal antibodies (mAbs) to cultures markedly suppressed the growth of GD2-expressing cells, as well as apoptosis induction [39]. Furthermore, Aixinjueluo et al. suggested that SCLC cell apoptosis induced by anti-GD2 mAbs may result from the dephosphorylation of FAK, a cytoplasmic tyrosine kinase with anti-apoptotic activity [40]. Reportedly, GT1b downregulates the expression of α5β1 integrin, caveolin-1, fibronectin, FAK, and ERK, while GT1b upregulates the expression of p53 and uPAR, resulting in downregulation of fibronectin-α5β1-integrin-ERK signaling in the A549 lung adenocarcinoma cell line. Thus, exogenous treatment with GT1b induces apoptosis in these cells [41].

### 2.7. Renal Urinary Cancer

Renal cell carcinoma (RCC) is the most common type of kidney cancer in adults. Reportedly, NEU3-silenced RCC, with high GD1a expression, demonstrates reduced FAK/AKT signaling and decreased drug resistance, invasive potential, and adhesion [42]. In human bladder cancer cells such as YTS-1, T24, 5637, and KK47 cells, exogenous addition of GM3 reduced cell proliferation, cell adhesion, and EGFR phosphorylation [43].

### 2.8. Other Types of Cancer

Notably, GD3 was first implicated in apoptosis in lymphoid and myeloid tumor cells, where the activation of the apoptosis-inducing CD95 death receptor (Fas) induced GD3 synthesis and accumulation in the mitochondrial membrane, resulting in the disruption of mitochondrial transmembrane potential and apoptosis in a caspase-independent manner [11]. NEU3 enhanced EGFR phosphorylation by downregulating GM3 in head and neck squamous cell carcinoma, leading to increased cell motility and invasion through increased matrix metalloproteinase (MMP)-9 expression [44].

## 3. Perspectives

**Figure 4:**
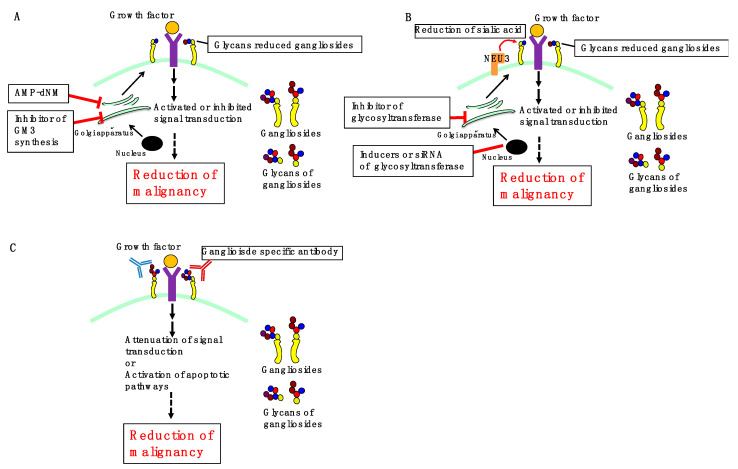
Three possible ganglioside-targeting strategies for cancer therapy. (**A**,**B**) Treatment with glucosylceramide synthase inhibitors such as AMP-dNM or specific inhibitors of GM3 synthase reduces glycans in gangliosides (**A**). Furthermore, treatment with an inhibitor of glycosyltransferases, or inducers or siRNA of glycosyltransferase or NEU3 reduces glycans in gangliosides (**B**). This affects the interaction of glycan-reduced gangliosides and receptors, activating or inhibiting signal, reducing malignancy. (**C**) Treatment with ganglioside-specific antibody contributes to attenuation of signal transduction or activation of apoptotic pathways, leading to reduced malignancy.

As mentioned above, ganglioside-induced changes in signaling have been associated with malignant behaviors of cancer. Therefore, the development of a therapeutic strategy targeting cancer-specific gangliosides may be valuable in cancer treatment. Three possible strategies targeting gangliosides (Figure 4), including (1) inhibition of ganglioside synthesis, (2) regulation of ganglioside expression by glycosyltransferases and/or sialidases, and (3) treatment with ganglioside-specific antibodies, are proposed in the following subsections.

### 3.1. Inhibition of Ganglioside Synthesis

Pharmacological depletion of gangliosides by using a specific inhibitor of glucosylceramide synthase has been performed in ganglioside-expressing cells to determine their endogenous significance. In a ganglioside-rich subline of B16 murine melanoma, ganglioside depletion following treatment with 1-phenyl-2-hexadecanoylamino-3-pyrrolidino-1-propanol markedly reduced tumor formation and metastasis in a mouse model of melanoma [73]. As described above, we previously demonstrated that treatment with the glucosylceramide synthase inhibitor, AMP-dNM, reduces ganglioside synthesis, which could inhibit TGF-β1 signaling and invasion in pancreatic cancer cells by possibly inhibiting the interaction between GM2 and TGFβRII [19]. Furthermore, it has been reported that AMP-dNM treatment improves glucose tolerance, reduces hepatic steatosis, and enhances insulin response in rodent models of type 2 diabetes by downregulating GM3 [74,75], and reduces the development of atherosclerosis in model mice, in which the ganglioside contribution remains unknown [76]. Thus, the effects of AMP-dNM-mediated targeting of gangliosides on age-related diseases, including diabetes and cancer, need to be elucidated. However, the inhibition of glucosylceramide synthesis by AMP-dNM reduces not only gangliosides but also other glycosphingolipids; therefore, it is necessary to develop ganglioside-specific inhibitors such as GM3 synthase inhibitors.

### 3.2. Regulation of Ganglioside Expression

In A431 epidermoid carcinoma cells, treatment with valproic acid reportedly increased GM3 synthase gene expression by approximately 8-fold. Furthermore, EGFR phosphorylation was reduced, while cell proliferation was inhibited following valproic acid treatment [32]. Inhibition of GD3 synthase can block the biosynthesis of b-series gangliosides, including GD3 and GD2, thus reducing proliferation, migration, and invasion of cancer cells. Accordingly, GD3 synthase may be a potential drug target for several cancers. Kwon et al. [77] revealed that the natural compound triptolide, a GD3 synthase inhibitor, inhibited cell proliferation by downregulating the expression of GD3 synthase in human melanoma SK-MEL-2 cells. Sarkar et al. [78] observed that triptolide inhibited tumor development by inhibiting the function of GD3 synthase in breast cancer. Transfection of the NEU3 sialidase gene into colon cancer cells inhibited apoptosis and was accompanied by increased Bcl-2 and decreased caspase expression. NEU3-transfected colon cancer cells exhibited marked accumulation of lactosylceramide. These results indicate that high NEU3 expression in cancer cells affords protection against programmed cell death, probably by modulating ganglioside expression. This finding indicates that NEU3 could be a possible target in colon cancer diagnosis and therapy [79]. As described in Section 2, increased NEU3 expression in cancer can be correlated with malignancy. Collectively, the development of glycosyltransferase inhibitors or inducers, NEU3 inhibitors, and nucleic acid drugs, including siRNA targeting glycosyltransferase or NEU3, can be expected as future cancer therapy.

### 3.3. Ganglioside-Specific Antibodies

Given the aberrant expression of specific gangliosides as signaling regulators in numerous cancers, antibodies against gangliosides, particularly anti-GD2 mAb, are currently being evaluated in preclinical studies or clinical investigations. Targeting tumor-associated gangliosides with antibodies can impact signaling pathways and induce cell death, including apoptosis [10,80]. For example, anti-proliferative and pro-apoptotic activities of anti-GD2 mAb 3F8 have been demonstrated in human melanoma cells [81]. In human NB cell lines, treatment with anti-GD2 mAb 14G2a attenuated PI3K/Akt/mTOR signaling, resulting in decreased cell viability [82]. The GD2-specific antibody hu14.18K322A is under investigation in a phase II trial in patients with NB [83]. Dinutuximab (a chimeric mAb targeting GD2 ganglioside) was approved by the U.S. Food and Drug Administration in 2015 and is currently used in a combination immunotherapeutic regimen for treating children with high-risk NB [84]. In human GD2 expressing mS melanoma, dinutuximab induced actin microfilament-dependent cell death [85]. In MG-63 and Saos-2 human osteosarcoma cells, cisplatin and anti-GD2 mAb 14G2a dose-dependently induced endoplasmic reticulum (ER) stress-associated apoptosis by activating the protein kinase RNA-like ER kinase (PERK) ER stress pathway by synergistically inducing phosphorylation and activation of PERK [86]. The humanized anti-ganglioside GM2 (GM2) antibodies, BIW-8962 and KM8927, induced high antibody-dependent cellular cytotoxicity and complement-dependent cytotoxicity in the GM2-expressing SCLC cell line SBC-3. Furthermore, humanized antibodies reportedly inhibit metastases and induce apoptosis in GM2-expressing SCLC cells and contribute to the prolonged survival of severe combined immunodeficient (SCID) mice [87]. A murine mAb (MAb-1) specific for GM3 showed antibody-dependent cellular cytotoxicity in two ovarian cancer cell lines (OVHM and ID8) and could be potentially employed as a therapeutic antibody against ovarian cancers in clinical trials [88]. To date, several vaccines have been developed based on glycans, including gangliosides [89,90]. However, the fact that glycans and glycopeptides are poorly immunogenic has posed significant challenges. Accordingly, addressing these issues will extend the application of ganglioside vaccines to cancer therapy.

In conclusion, we presented the possibility of cancer therapy that targets gangliosides, which are known to contribute to the regulation of cancer cell signaling. In recent years, near-infrared photoimmunotherapy (NIR-PIT) targeting cancer cell antigens and induction of senescence in cancer cells have gained momentum [91,92,93]. In the future, novel therapies for cancer cells expressing gangliosides, such as NIR-PIT using ganglioside-specific antibodies, as well as senolysis of senescence-induced cancer cells through ganglioside-mediated signaling, could be employed as promising therapeutic strategies. Therefore, further ganglioside-targeting research is needed for the effective development of suitable cancer treatments.

## Figures and Tables

**Table 1:** Signal regulation of endogenous or exogenous gangliosides in cancer cells.

Types of Cancer	Types of Cells	Types of Gangliosides	Functional Roles	References
Hepatocellular Carcinoma	human hepatoma HepG2	GD1a	Inhibition of HGF/c-Met signaling	[13]
	human hepatoma HepG2	GD3	Inhibition of NF-kB signaling	[14]
	mouse hepatocellular carcinoma Hepa1-6	GM3 or GM2 and GM3	Inhibition of HGF signaling in CD82-upregulated cells	[15]
	mouse hepatic cancer cell lines (Hca/A2, Hca/16A3, and Hepa1-6)	GM3	Promotion of HGF/c-Met and PI3K/Akt signaling	[16,17]
	mouse hepatocellular carcinoma Hepa1-6	GM3	Inhibition of EGF signaling	[17]
	mouse ascites hepatoma cell line HcaF	GM3	Inhibition of phosphorylation of Akt and EGFR	[18]
Pancreatic Cancer	human pancreatic cancer MIA PaCa-2	GM2	Promotion of TGF-b1 signaling	[19]
Colorectal Cancer	human colon cancer HCT116	GM3	Inhibition of the PI3K/Akt/MDM2 signaling	[20]
	human colon cancer HCT116	GM3	Promotion of oxidative stress-mediated mitochondrial pathway	[21]
	human colon cancer HCT116 and HT-29	GM3	Inhibition of Wnt/b-catenin signaling	[22]
Neural and Brain Cancer	human neuroblastoma cell line NBL-W	GT1b, GM3, or GD1a	Inhibition of EGF signaling	[23]
	neuroblastoma	GM1	Activation of the TrkA receptor	[24]
	human glioma	GM1	Inhibition of PDGF signaling	[25]
	human glioma	GD3	Promotion of PDGF signaling	[26]
	glioma cell line U-251MG	GD3 and GD2	Promotion of ERK1/2 and Akt pathway	[27]
	glioblastoma multiform cell line	GD3	Promotion of c-Met signaling	[28]
Skin Cancer	human melanoma	GD3	Activation of Src family kinase	[29]
	human melanoma	GD3	Promotion of HGF signaling	[30]
	human melanoma	GD3	Promotion of p130Cas and paxillin pathway	[31]
	human epidermoid carcinoma A431	GM3	Inhibition of EGF signaling	[32]
Sex Hormone-Related Cancer	breast cancer cell MDA-MB231	GD2	Promotion of c-Met signaling	[33]
	breast cancer cell MDA-MB468	GD3	Promotion of EGF signaling	[34]
	triple-negative breast cancer	GD2	Promotion of FAK-Akt-mTOR signaling	[35]
	human breast cancer MCF-7	GD1b	Activation of apoptotic pathway	[36]
	prostate cancer PC-3 and LNCaP	GM3	Inhibition of EGF signaling	[37]
Bone Cancer	mouse osteosarcoma cell variant FBJ-LL	GD1a	Inhibition of HGF/c-Met signaling	[13]
	human osteosarcoma	GD3 and GD2	Promotion of p130Cas, FAK and paxillin pathway	[38]
Lung Cancer	small cell lung cancer	GD2	Inhibition of growth and induction of apoptosis by anti-GD2 mAb	[39]
	small cell lung cancer	GD2	Promotion of FAK pathway	[40]
	A549 lung adenocarcinoma	GT1b	Inhibition of fibronectin-a5b1-integrin-ERK signaling	[41]
Renal Urinary Cancer	human renal cell carcinoma cell	GD1a	Inhibition of the FAK/Akt signaling	[42]
	human bladder cancer YTS-1, T24, 5637, and KK47	GM3	Inhibition of EGF signaling	[43]
Other Types of Cancer	lymphoid and myeloid tumor cells	GD3	Activation of CD95-mediated apoptotic pathway	[11]
	squamous carcinoma HSC-2 and SAS	GM3	Inhibition of EGF signaling	[44]

EGF, epidermal growth factor; EGFR, epidermal growth factor receptor; HGF, hepatocyte growth factor; PDGF, platelet-derived growth factor; FAK, focal adhesion kinase; ERK1/2, extracellular signal-regulated kinase 1/2.
